# Supplementary material for: Changes in Saliva Analytes Associated with Lameness in Cows: A Pilot Study
Source: Animals (Basel). 2020 Nov 9;10(11):2078. doi: 10.3390/ani10112078 (PMC7696794; doi:10.3390/ani10112078)
Supplement: Supplementary file 1 [file animals-10-02078-s001.pdf]

animals

MDPI

Article

Changes in Saliva Analytes Associated with Lameness in Cows: a Pilot Study

María D. Contreras-Aguilar [1], Pedro Javier Vallejo-Mateo [2], Rasa Želvytė [3], Fernando Tecles [1,*] and Camila Peres Rubio [1]

[1] Interdisciplinary Laboratory of Clinical Analysis of the University of Murcia (Interlab-UMU), Veterinary School, Campus Mare Nostrum, University of Murcia, 30100 Murcia, Spain; mariadolores.contreras@hotmail.com (M.D.C.-A.); ftecles@um.es (F.T.); camila.peres@um.es (C.P.R.)
[2] Department of Animal Medicine and Surgery, Veterinary School, Campus Mare Nostrum, University of Murcia, 30100 Murcia, Spain; pedroja512@hotmail.com
[3] Department of Anatomy and Physiology, Research Center of Digestive Physiology and Pathology, Veterinary Academy, Lithuanian University of Health Sciences, Tilzes str. 18, LT-47181 Kaunas, Lithuania; Rasa.Zelvyte@lsmuni.lt
* Correspondence: ftecles@um.es

Received: 24 September 2020; Accepted: 3 November 2020; Published: date

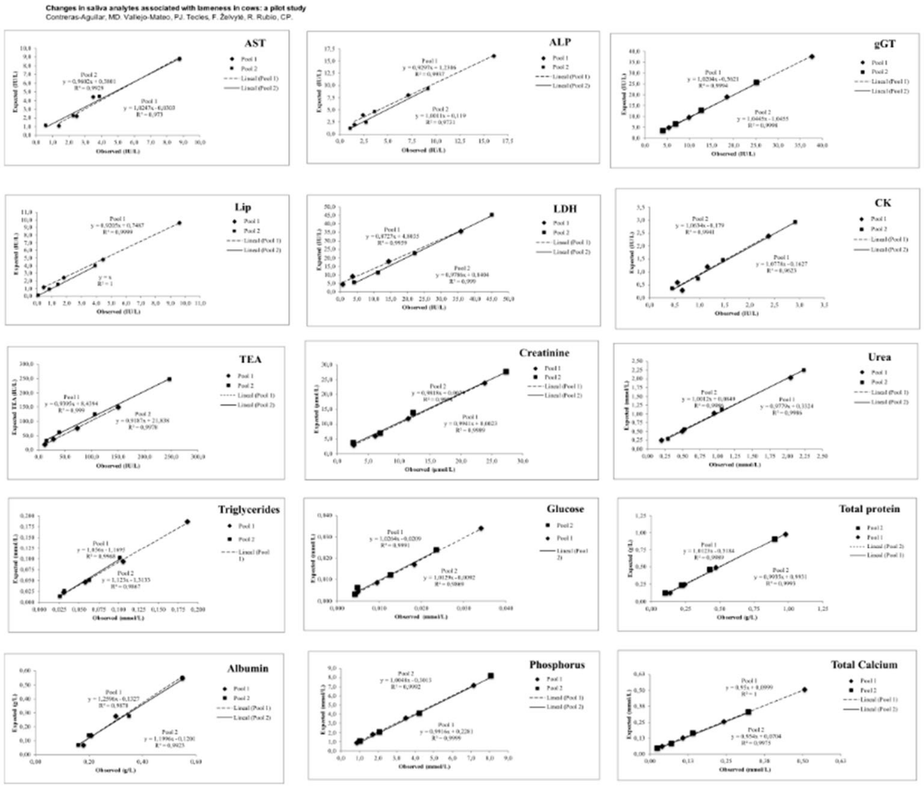

Figure S1. Linearity under dilution in saliva of aspartate aminotransferase (AST), alkaline phosphatase (ALP), γ-glutamyl transferase (gGT), lipase (Lip), lactate dehydrogenase (LDH), creatine kinase (CK), total esterase (TEA), creatinine, urea, triglycerides, glucose, total protein, albumin, phosphorus, and total calcium. Linearity under dilution study in two pools of saliva from three specimens of saliva each. The 'x' expressed activity or concentration measured and 'y' expected level at the particular dilution. R2 = coefficient of determination of linear correlation.
